# Three measures of physical rehabilitation effectiveness in elderly patients: a prospective, longitudinal, comparative analysis

**DOI:** 10.1186/s12877-015-0138-5

**Published:** 2015-10-29

**Authors:** Dolores Sánchez-Rodríguez, Ramon Miralles, Josep M. Muniesa, Sergio Mojal, Anna Abadía-Escartín, Olga Vázquez-Ibar

**Affiliations:** Geriatrics Department, Parc de Salut Mar Centre Fòrum Hospital del Mar (Llull, 410), Universitat Autònoma, Barcelona, (08019) Spain; Physical Medicine and Rehabilitation Department, Parc de Salut Mar Hospital de l´Esperança (Sant Josep de la Muntanya, 12), Universitat Autònoma, Barcelona, (08024) Spain; Biomedical Research Methods Consultant, Hospital del Mar Medical Research Institute (IMIM) (Doctor Aiguader 88), Barcelona, (08003) Spain; Geriatrics Department, Parc de Salut Mar Hospital de l´Esperança (Sant Josep de la Muntanya 12), Barcelona, (08024) Spain

**Keywords:** Geriatric rehabilitation, Functional recovery, Elderly, Rehabilitation impact index, Post-acute

## Abstract

**Background:**

Rehabilitation success is measured by instruments that assess performance of activities of daily living. Guidelines on the use and choice of these instruments are lacking. The present study aimed to analyse prognostic indicators of physical rehabilitation effectiveness in elderly patients according to three rehabilitation impact indices.

**Methods:**

Prospective, longitudinal study in a post-acute care unit. The study included rehabilitation-eligible deconditioned elderly in-patients prospectively admitted to post-acute care (*n* = 685, aged 83.2 ± 8.3 years, mean length of stay 15 ± 9.2 days).

Data Collection: Premorbid health status variables (PHSV): age, sex, comorbidity (Charlson index), medical history (heart failure, pulmonary disease, cerebrovascular disease, dementia), previous living situation and pre-admission functional status (premorbid Lawton and Barthel indices). Admission health status variables (AHSV): main diagnoses, referral source, physical (Barthel-adm) and cognitive function (Pfeiffer test), undernutrition and dysphagia.

Outcome Measures: Absolute functional gain (AFG, admission-to-discharge Barthel change), relative functional gain (RFG, achieved percentage of potential gain) and rehabilitation efficiency index (REI, AFG over length of stay). Univariate analysis considered these parameters, along with PHSV and AHSV. Multivariate logistic regression analysis was performed for AFG ≥20, RFG ≥35 % and REI ≥ 0.50.

**Results:**

Greater AFG was associated with 14 variables, 8 PHSV (57.1 %) and 6 AHSV (42.8 %); greater RFG with 9 variables, 3 PHSV (33.3 %) and 6 AHSV (66.6 %); and REI with 9 variables, 4 PHSV (44.4 %) and 5 AHSV (55.5 %). Mean AFG value was 34.5 ± 15.8 in patients who achieved complete recovery (RFG 100 %, *n* = 189, 27.5 %) and 35.3 ± 15.0 (*p* = 0.593) in the remaining patients (*n* = 311, 45.4 %). In multivariate analysis, only Barthel-adm was related to all three rehabilitation impact indices.

**Conclusions:**

Both premorbid and acute-process variables have a greater impact on AFG and REI, compared to RFG. Although AFG gives information about the degree of reduction in dependence, it does not provide clinical information about post-rehabilitation functional status (mean AFG values did not differ between patients with and without complete recovery). A future implication for evaluating rehabilitation effectiveness in elderly patients is to recommend RFG corrected by premorbid Barthel score, which is less affected by previous health conditions, as the optimum method to assess the degree to which maximum potential improvement was achieved.

**Electronic supplementary material:**

The online version of this article (doi:10.1186/s12877-015-0138-5) contains supplementary material, which is available to authorized users.

## Introduction

Elderly patients admitted to hospitals, whether for acute or decompensated chronic disease, often present with loss of functional capacity that can lead to dependency and disability [1–3]. This complicates recovery from an acute episode, impeding return to their previous living situation and requiring physical rehabilitation and additional health care resources post-discharge [4–8]. Many predictors of functional recovery have been described in elderly patients [9, 10] but varied according to patient complexity and population characteristics, which also may affect functional improvement and the time required to achieve it. Rehabilitation success is measured by the scores achieved on instruments that assess performance of activities of daily living. Rehabilitation Impact Indices (RIIs) are composed of the scores on these instruments, and include functional status before admission, upon admission to rehabilitation and at discharge, as well as the length of the rehabilitation programme.

One of the best-known RIIs is “absolute functional gain” (AFG) [9, 11, 12], which assesses the difference between admission and discharge functional scores. Other authors use “relative functional gain” (RFG) [9, 10], also called the Heinemann index or Montebello Rehabilitation Factor score [9, 11, 13], which expresses functional recovery as a percentage of the maximum potential improvement. Recently, some authors have labelled this parameter more simply as “rehabilitation effectiveness” [14]. Maximum potential improvement is the return to an individual’s premorbid functional score. In previously healthy subjects, this should coincide with the maximum score on the evaluation instrument; in elderly patients, premorbid status may not reach this maximum score. In these cases, a modified parameter has been used, and called “the RFG corrected for premorbid functional status” or the “corrected Heinemann index” [15]. Finally, the “rehabilitation efficiency index” (REI) expresses the average increase *per day* in a functional assessment score; this outcome considers speed of recovery [9, 14, 16, 17].

In the elderly, previous functional status may reflect comorbidities or other prior conditions that affect health status. In these cases, the addition of one or more new disabilities associated with a new clinical condition further reduces the patient’s functional status at the start of the rehabilitation. Therefore, RIIs that are calculated on the basis of the absolute functional values at the start of the rehabilitation programme, such as the AFG, may be more influenced by variables that affect previous health status. On the other hand, RIIs that can be calculated using recent loss of functional capacity, such as the RFG, may evaluate only the loss related to the acute process and therefore would be less affected by the patient’s previous health status.

The specific aims of this study were to assess the application of these RIIs in hospitalized elderly patients with impaired functional capacity, to identify differences between the results obtained by the three RIIs, and to discuss ways to ensure their accurate and appropriate use.

## Methods

### Study population and setting

Patients aged 75 years and older, with recent loss of functional capacity due to acute disease, decompensated chronic disease, or surgical procedure were included. The interdisciplinary care team conducted a comprehensive geriatric assessment [18] for each patient within 72 h of admission and developed an individualized care plan.

Patients were eligible for referral to the rehabilitation programme if medically stable (absence of acute infection, absence of symptomatic worsening of chronic disease and absence of acute confusion) and judged able to participate in physical therapy, as indicated by initial comprehensive geriatric assessment performed by an interdisciplinary care team. A further inclusion criterion was the potential to achieve rehabilitation goals, manage medical conditions and develop a discharge plan in approximately two weeks, based on pre-admission geriatric assessment of the patient’s clinical characteristics [4, 6, 8]. The exclusion criterion was absence of functional decline, based on the comprehensive assessment; these patients were admitted for transitional care and monitoring, completion of medical treatment, or medication management but were not referred for any specific rehabilitation therapy.

As part of the interdisciplinary care plan developed upon admission to the programme, a rehabilitation specialist prepared a plan to improve functional capacity, mobility, and independence in basic activities of daily living. Initially, rehabilitation involved in-bed therapy and active-assisted movement of peripheral and respiratory muscles, including isometrics exercises, depending on patient tolerance and cooperation. As patients progressed, the programme included training on transfers and re-education on assisted gait. Finally, patients with highly favourable outcomes received training on stairs and a gait circuit. Patients were discharged on the basis of clinical course, and home-based physical therapy was considered when indicated.

### Data collection

During the 18-month study period, 753 patients were admitted to the unit. All patients consented to study participation and received a comprehensive geriatric assessment. The standard geriatric assessment of each individual includes the recording of premorbid health status variables (PHSV), an initial group of variables related to health and/or functional status before the health incident that lead to hospital admission, as well as admission health status variables (AHSV), which reflect the deterioration in health and/or functional status at the time of admission to the rehabilitation programme and therefore indirectly show the severity of the acute process that led to admission. The PHSV data include age, sex, comorbidity (Charlson index [19]), medical history (heart failure, pulmonary disease, cerebrovascular disease and dementia), previous living situation (alone, with family, assisted living centre) and functional status before hospital admission [instrumental and basic activities of daily living measured by Lawton [20] and Barthel index [21] (BI-premorbid)]. To add clinical meaning to the analysis of the BI-premorbid parameter, the present study divided the scores into four categories (0–20, 21–40, 41–60, and 61–100, from most to least dependent), as previously determined by Granger et al. [21].

The AHSV include main diagnoses at admission (ICD categories), referral source, functional status at admission (BI-adm), cognitive status (Pfeiffer test [22]), dysphagia (clinical evaluation) and undernutrition. This last parameter was defined by low values in protein, albumin and/or total cholesterol [(Laboratory reference values of our institution were: protein (6–8.3 mg/dL), albumin (3.8-5.1 mg/dL) and cholesterol (120–200 mg/dL)].

BI-adm was assessed within 72 h of admission in all cases as part of the systematic comprehensive geriatric assessment. BI-premorbid was also routinely completed using anamnesis and patient interviews, confirmed by family and previous medical reports as needed. The index was again administered 24 h prior to discharge (BI-disch). All these measurements were recorded and discussed by the interdisciplinary team, as part of the daily clinical activity of our rehabilitation ward. Finally, discharge destination and social worker intervention (assessment, resource information for caregivers and discharge planning) were recorded.

### Functional status and RIIs

The AFG score was calculated to reflect the BI change post-rehabilitation [9, 11, 12] (BI-disc – BI-adm). An AFG ≥20 was considered a clinically important difference [12]. The RFG corrected with premorbid functional status was calculated as follows: [RFG = (BI ‐ disc – BI ‐ adm)/(BI ‐ premorbid – BI ‐ adm) × 100]. RFG shows the percentage of the premorbid functional capacity recovered at discharge, in relation to what had been lost at admission to rehabilitation [9, 11, 15]. A RFG ≥35 % means that the patient has recovered at least one third of the functional loss observed; therefore, some authors have described this value as a point beyond which rehabilitation can be considered clinically effective [10, 17, 23].

The present study considered four patient groups based on the published RFG value and clinical criteria. Group I (RFG = 0) included patients who died, lost functional capacity while in the unit or were urgently transferred to hospital for an acute event or worsening clinical status; negative and undetermined RFG values were assigned to this group for purpose of analysis. Rehabilitation effectiveness was considered low to moderate in Group II (RFG 1–34 %); high in Group III (RFG 35–99 %), and as achieving complete recovery in Group IV (RFG 100 %) [17, 23]. Finally, REI was calculated as follows: REI = (BI-disc – BI-adm) / days of stay. This index shows functional gain (BI points gained) per day [9, 17]. A negative value indicates worsened functional status during the rehabilitation unit stay; 0 to 0.49 shows low rehabilitation efficiency; 0.50 to 1 reflects moderate rehabilitation efficiency; and >1 indicates high efficiency [17, 23–25]. A REI ≥0.50 was considered a clinically important difference [17].

## Statistical analysis

Univariate analysis was used to find associations between patient characteristics and results on the three different RIIs. Qualitative variables were compared by Chi-square or Fisher exact test, as appropriate, and quantitative variables by Student *T* test or single-factor analysis of variance (ANOVA) with Tukey multiple comparison correction. Finally, variables significantly associated with each RII were included in a binary multivariate logistic regression to obtain an optimal model for predicting recovery, based on the cut-off value established for each dependent variable. Given that quantitative variables behave differently according to the dependent variable analysed, different cut-off values were compared and tested with the dependent variables (AFG ≥20, RFG ≥35 % and REI ≥0.50, respectively) in order to assess linearity or to find the best cut-off points before including them in the model. For each model, OR (95 %CI), discrimination power and calibration were calculated. Analyses were done using SPSS 18.0 (IBM Corporation, SPSS, INC., Chicago, IL, USA).

## Ethics

National and international research ethics guidelines were followed, including the Deontological Code of Ethics, Declaration of Helsinki (Fortaleza 2013) and Spain’s confidentiality law concerning personal data (Ley Orgánica 15/1999, 13 December). Detailed, understandable information was provided to patients and family members, and oral informed consent to participate was obtained from all potential participants during their hospital stay, before beginning the post-acute unit’s comprehensive geriatric assessment. In patients with dementia, oral informed consent was obtained from the main caregiver. The institution’s Clinical Ethics Committee approved the study and the informed consent process used (*Comité Etico de Investigación Clínica Parc de Salut Mar*: reference number 6370/I). STROBE Guidelines for reporting observational research cohorts were followed [26] (Additional file 1).

## Results

After completion of the comprehensive assessment, 68 patients were excluded from analysis because there was no evidence of functional decline. The characteristics of the 685 included patients are shown in Table 1.

**Table 1:** Descriptive data of patients (*n* = 685)

Admission data	
Variables	n (%)
Premorbid health status variables (PHSV)	
Sex	
Male	290 (42.3 %)
Female	395 (57.7 %)
Age (years)^a^	83.2 (±8.3)
Age	
<85 years	348 (50.8 %)
≥85 years	337 (49.2 %)
Medical history	
Heart failure	170 (24.8 %)
Pulmonary disease	211 (30.8 %)
Cerebrovascular disease	135 (19.7 %)
Dementia	332 (48.5 %)
Comorbidity (Charlson index)	
0–1 (absence of comorbidity)	183 (26.7 %)
2 (low comorbidity)	143 (20.9 %)
3 or more (high comorbidity)	359 (52.4 %)
Previous functional capacity, instrumental activities (Lawton index, points)^b^	
0	272 (40.5 %)
1 a 5	247 (36.9 %)
6 a 8	151 (22.6 %)
Previous functional capacity (premorbid Barthel index, points)	
Mean (±SD)^a^	71.0 (±25.1)
Median [P_25_-P_75_]	77 [52–95]
0–20 (total dependence)	27 (3.9 %)
21–40 (severe dependence)	64 (9.3 %)
41–60 (moderate dependence)	141 (20.6 %)
61–100 (slight dependence)	453 (66.1 %)
Previous living situation	
Alone	215 (31.4 %)
With family	357 (52.1 %)
With others	46 (6.7 %)
Residential/assisted living centre	67 (9.8 %)
Admission health status variables (AHSV)	
Referral source	
Acute care geriatric unit	439 (64.1 %)
Other medical-surgical specialties	246 (35.9 %)
Diagnosis during hospitalization for acute care	
Neurological	72 (10.51 %)
Respiratory	311 (45.4 %)
Cardiological	186 (27.2 %)
Infections (except respiratory and central nervous system)	253 (36.9 %)
Dyselectrolytemia	236 (34.5 %)
Endocrinopathy	52 (7.6 %)
Musculoskeletal	152 (22.2 %)
Confusion syndrome	305 (44.5 %)
Other	169 (24.7 %)
Functional capacity at admission (Barthel index, points)	
Mean (±SD)^a^	28.00 (±19.90)
Median [P_25_-P_75_]	25 [15–41]
0–20 (total dependence)	302 (44 %)
21–40 (severe dependence)	212 (31 %)
41–60 (moderate dependence)	126 (18.4 %)
61–100 (slight dependence)	45 (6.6 %)
Cognitive deterioration at admission (Pfeiffer test, error)^c^	
Mean (±SD)^a^	4.19 (±3.04)
Median [P_25_-P_75_]	4 [2–6]
Little or none (0–2)	233 (34.6 %)
Moderate (3–7)	312 (46.4 %)
Severe (8–10)	128 (19 %)
Dysphagia at admission	217 (31.9 %)
Undernutrition at admission	624 (91.4 %)
Discharge and evolution data	
Variables	n (%)
Functional capacity at discharge (Barthel index, points)	
Mean (±SD)^a^	57.80 (±27.31)
Median [P_25_-P_75_]	62 [37–80]
0–20 Total dependency	145 (21.2 %)
21–40 Severe dependency	87 (12.7 %)
41–60 Moderate dependency	130 (19 %)
61–100 Slight dependency	323 (47.1 %)
Discharge destination	
Home	437 (63.8 %)
Residential / Nursing Home	136 (19.9 %)
Intermediate care / Long Term care	52 (7.6 %)
Deaths or patients transferred to acute care	60 (8.8 %)
Length of stay (days)^a^	15 (±9.2)
Absolute Functional Gain (AFG) (Barthel index, points)^a^	25 (±23.1)
Relative Functional Gain (RFG) (%)^a^	61.7 (±37.7)
Rehabilitation Efficiency index (REI) (BI points / day)^a^	2 (±2.8)

^a^Values expressed as mean ± standard deviation. SD: standard deviation

^b^Evaluated in 670 patients

^c^Evaluated in 673 patients

AFG = Barthel index at discharge (BI ‐ disc)–Barthel index at admission (BI ‐ adm) (see text)

RFG = [(BI ‐ disc–BI ‐ adm)/(BI ‐ premorbid–BI ‐ adm) × 100] (see text)

REI = [(BI ‐ disc–BI ‐ adm)/length of stay] (see text)

At discharge (Table 1), patients had achieved major improvements in functional capacity (BI-adm = 28.00 ± 19.90; BI-disch = 57.80 ± 27.31), with a mean AFG increase of 25 BI points and a mean value of 61.7 % on the RFG and 2 points on the REI. At discharge, 21.2 % of patients were classified as having severe dependency; it should be noted that patients who died, were rehospitalized due to complications or were discharged to long-term care facilities were included in this group. Table 2 shows the patient groups by RFG and the respective AFG and REI values. Mean AFG and REI values differed significantly between the four patient groups, classified according to the level of change achieved in the RFG; the exception was the AFG comparison in Groups III and IV, which showed similar mean values.

**Table 2:** Relative Functional Gain (RFG), Absolute Functional Gain (AFG) and Rehabilitation Efficiency index (REI) (*n* = 685)

Group	Number of patients N (%)	Relative Functional Gain (RFG)	Absolute Functional Gain (AFG)^a,c^	Rehabilitation Efficiency index (REI)^a,c^
Group I	117 (17.2 %)	0 (negative, indeterminate^b^)	−9.8 (±19.1)	−1.4 (±3.7)
Group II	68 (9.9 %)	1–34 % (low-moderate)	11.2 (±6.2)	0.8 (±0.6)
Group III	311 (45.4 %)	35–99 % (elevated)	35.2 (±15.0)	2.7 (±1.7)
Group IV	189 (27.5 %)	100 % (complete recovery)	34.5 (±15.8)	3.3 (±2.2)

^a^Values expressed as mean ± standard deviation (REI ≥1 indicates high efficiency of the rehabilitation process)

^b^For purposes of analysis, negative or indeterminate values resulting from calculations were converted to 0. (Patients who died, lost functional capacity during their stay in the unit or were transferred to acute care hospitals for an acute event or worsening clinical status are included in this group)

^c^There were significant differences in mean values between all the groups (*p* < 0.001), except for the AFG between groups III and IV (*p* = 0.593)

As summarized in Table 3 by RII and in Table 4 by primary diagnosis, when effectiveness of the rehabilitation process was evaluated using the AFG, 14 variables were associated with greater AFG, eight of them (57.1 %) from the PHSV group (age < 85y, absence of previous history of heart failure, absence of previous history of pulmonary disease, absence of previous history of dementia, absence of comorbidity; higher premorbid Lawton and BI scores, living alone) and six (42.8 %) from the AHSV group (referral source different than acute care geriatric unit, lower score in BI-adm, better cognitive function, absence of dysphagia, absence of respiratory diagnosis, main diagnosis of endocrinopathy at admission). When increased RFG values were used to evaluate effectiveness, nine variables were significantly related, three of them (33.3 %) from the PHSV group (age under 85y, absence of previous history of dementia, higher scores in previous Lawton index) and six (66.6 %) from the AHSV group (higher BI-adm scores, better cognitive function, absence of dysphagia, absence of undernutrition, absence of respiratory diagnosis, main diagnosis of endocrinopathy at admission). Finally, the highest REI values also were associated with nine variables, four (44.4 %) from the PHSV group (absence of previous history of dementia, absence of comorbidity, higher BI-premorbid scores, living alone) and five (55.5 %) from the AHSV group (BI-adm score 41–60, absence of dysphagia, absence of undernutrition, absence of respiratory diagnosis, main diagnosis of endocrinopathy at admission). As shown in Tables 3 and 4, only five of the total 24 variables studied (20.8 %) were related to all three RIIs (history of dementia, respiratory disease, dysphagia, main diagnosis of endocrinopathy, functional capacity at admission).

**Table 3:** Rehabilitation Impact Indices, premorbid and at admission health status variables (*n* = 685)

Variables	Number	Absolute Functional Gain (AFG)	Relative Functional Gain (RFG)	Rehabilitation Efficiency index (REI)
Previous health status variables (PHSV)				
Sex				
Male	290	25.4 (±25.6)	59.3 (±38)	1.9 (±3.4)
Female	395	24.7 (±21.2)	63.5 (±37.3)	2.1 (±2.2)
p-value		0.407	0.112	0.549
Age				
<85 years	348	26.4 (±24.4)	64.7 (±38.3)	2.1 (±3.1)
≥85 years	337	23.5 (±21.6)	58.7 (±36.8)	1.9 (±2.5)
p-value		0.026	0.009	0.161
Medical history				
Heart failure	170	20.5 (±24.6)	62.8 (±40.3)	1.6 (±3.2)
No heart failure	515	26.5 (±22.5)	61.4 (±36.8)	2.1 (±2.6)
p-value		0.013	0.250	0.228
Pulmonary disease	211	20.9 (±26.1)	59.5 (±40.5)	1.7 (±3.1)
No pulmonary disease	474	26.8 (±21.4)	62.7 (±36.3)	2.1 (±2.6)
p-value		0.017	0.778	0.101
Cerebrovascular disease	135	22.9 (±23.1)	56.8 (±39.6)	1.6 (±2.8)
No cerebrovascular disease	550	25.5 (±23.1)	62.9 (±37.1)	2.1 (±2.8)
p-value		0.155	0.120	0.139
Dementia	332	22.9 (±20)	57.5 (±38.7)	1.9 (±2.6)
No dementia	353	27 (±25.6)	65.7 (±36.2)	2.1 (±3)
p-value		<0.001	0.018	0.009
Comorbidity (Charlson index)				
≤1 (absence of comorbidity)	183	29.7 (±20.6)	65.9 (±33.3)	2.6 (±2.5)
2 (low comorbidity)	143	25.5 (±23.1)	59.3 (±39)	2 (±2.9)
3 or more (high comorbidity)	359	22.4 (±24)	60.6 (±39.1)	1.7 (±2.9)
p-value		0.002	0.647	0.003
Previous functional capacity, key activities (Lawton index, points)^a^				
0				
1–5	272	19.4 (±19.4)	56.9 (±40.3)	1.7 (±2.5)
6–8	247	26.3 (±23.7)	63.4 (±36.8)	2.2 (±2.7)
p-value	151	32.1 (±25.9)	66.9 (±33.1)	2.2 (±3.4)
		<0.001	0.019	0.111
Previous functional capacity (premorbid Barthel index, points)				
0–20	27	15.7 (±13.4)	51.3 (±50.4)	1.7 (±2)
21–40	64	16.3 (±12.9)	70 (±38.4)	1.4 (±1.2)
41–60	141	19.4 (±19.6)	57.7 (±41.3)	1.6 (±2.4)
61–100	453	28.5 (±24.9)	62.4 (±35.3)	2.2 (±3.1)
p-value		<0.001	0.074	0.023
Previous living situation				
Alone	215	30.8 (±23.9)	64.7 (±34.8)	2.2 (±2.8)
With family	357	23.5 (±22.5)	62.1 (±38.5)	1.9 (±2.7)
With others	67	22.2 (±20.2)	58.2 (±37.6)	1.9 (±2.7)
Residential/assisted living centre	46	13.2 (±21.4)	49.8 (±42.4)	1.6 (±2.9)
p-value		<0.001	0.317	0.015
Admission health status variables (AHSV)				
Source of referral				
Acute care geriatric unit	439	23.2 (±21.5)	60.1 (±38.2)	2.1 (±2.4)
Other medical-surgical specialties	246	28.2 (±25.6)	64.7 (±36.5)	1.9 (±3.4)
p-value		<0.001	0.210	0.343
Functional capacity at admission (Barthel index, points)				
0–20	302	22.8 (±22.4)	50 (±39.4)	1.6 (±3)
21–40	212	28.2 (±24.7)	68.1 (±34)	2.1 (±2.7)
41–60	126	28.9 (±20.1)	73.9 (±30.8)	2.8 (±2.5)
61–100	45	14.1 (±23.5)	77.7 (±36.2)	2 (±2.4)
p-value		<0.001	<0.001	0.001
Cognitive deterioration at admission (Pfeiffer test, error) ^b^				
Little or none (0–2)	233	28.8 (±24.3)	69 (±35.1)	2.3 (±2.8)
Moderate (3–7)	312	23.9 (±22.8)	61.6 (±37.4)	2 (2 ± .6)
Severe (8–10)	128	22 (±20.5)	50.6 (±39)	1.8 (±2.2)
p-value		0.012	<0.001	0.148
Dysphagia				
Yes	217	18.5 (±22.7)	49.9 (±40.8)	1.4 (±3.2)
No	464	28.1 (±22.6)	67.4 (±34.7)	2.3 (±2.4)
p-value		<0.001	<0.001	<0.001
Undernutrition				
Yes	624	24.7 (±23.4)	61 (±37.8)	1.9 (±2.7)
No	59	28.6 (±18.8)	71.5 (±33.6)	2.8 (±2.3)
p-value		0.322	0.041	0.031

^**a**^Evaluated in 670 patients

^b^Evaluated in 673 patients

**Table 4:** Rehabilitation Impact Indices and primary diagnosis upon admission at post-acute geriatric care unit (*n* = 685)

Diagnosis	Number	Absolute Functional Gain (AFG)	Relative Functional Gain (RFG)	Rehabilitation Efficiency index (REI)
Neurological				
Yes	72	26.2 (±21.8)	53.8 (±39)	2 (±1.8)
No	613	24.9 (±23.3)	62.7 (±37.4)	2 (±2.9)
p-value		0.966	0.068	0.420
Respiratory				
Yes	311	20.9 (±24.5)	55.7 (±39.4)	1.6 (±3.1)
No	374	28.4 (±21.4)	66.7 (±35.4)	2.3 (±2.5)
p-value		<0.001	<0.001	0.002
Cardiological				
Yes	186	23.4 (±25.7)	61.5 (±39.3)	1.8 (±2.8)
No	499	25.6 (±22.1)	61.8 (±37.1)	2.1 (±2.8)
p-value		0.409	0.763	0.363
Other infections				
Yes	253	24.8 (±23.7)	60.5 (±36.8)	2 (±2.7)
No	432	25.1 (±22.8)	62.5 (±38.2)	2 (±2.8)
p-value		0.896	0.385	0.546
Dyselectrolytemia				
Yes	236	24.9 (±23.2)	62.7 (±37.2)	2.1 (±2.6)
No	449	25.1 (±23.1)	61.2 (±37.9)	2 (±2.9)
p-value		0.891	0.541	0.464
Endocrinopathy				
Yes	52	32.6 (±19.7)	73.3 (±32.3)	2.8 (±2.1)
No	633	24.4 (±23.3)	60.8 (±37.9)	1.9 (±2.8)
p-value		0.015	0.031	0.014
Musculoskeletal				
Yes	152	23.8 (±22.5)	58 (±38)	1.6 (±2.8)
No	533	25.3 (±23.3)	62.8 (±37.5)	2.1 (±2.8)
p-value		0.339	0.149	0.082
Confusion Syndrome				
Yes	305	25.9 (±19)	63.1 (±37.4)	2.1 (±2.4)
No	380	24.3 (±26)	60.6 (±37.8)	1.9 (±3.1)
p-value		0.607	0.260	0.995
Others				
Yes	169	26.3 (±25.1)	62.4 (±37.9)	1.9 (±2.9)
No	516	24.6 (±22.5)	61.5 (±37.6)	2 (±2.7)
p-value		0.252	0.796	0.747

Differences between groups for each of the three RIIs are shown in Table 3. On the AFG, the BI-adm 61–100 group had a significantly lower mean score than the other three groups; on the RFG, the BI-adm 0–20 group differed significantly from the other three, and on the REI the only significant difference was observed between the BI-adm 0–20 and 41–60 groups.

In multivariate analysis, four variables were related to achieving a minimal clinically important difference in AFG (≥20): Charlson index ≤3, absence of dysphagia, instrumental activities of daily living (Lawton index) and a BI-adm score between 11 and 50. In patients with an AFG >50, a ceiling effect was observed. These variables were included in both the PHSV and AHSV analysis. In multivariate results, absence of a respiratory diagnosis and a BI-adm score >11 were associated with a higher probability of high, or even complete, functional recovery (RFG ≥35 %) and the same two variables together with the absence of dysphagia were associated with a clinically important difference in REI (≥0.50). Therefore, BI-adm was the only variable related to all three RIIs (Table 5).

**Table 5:** Variables related to a high effectiveness of rehabilitation in three multivariate logistical regression models

Variable	OR (95 % CI)	*p*-value
Absolute functional gain ≥20		
Charlson index ≤3	1.52 (1.08–2.12)	0.015
Lawton index		
0	ref.	
1–5	1.60 (1.07–2.38)	0.022
6–8	2.38 (1.42–4.02)	0.001
Functional capacity at admission		
Barthel index ≤10	ref.	
Barthel index 11–50	2.68 (1.75–4.11)	<0.001
Barthel index >50	0.96 (0.51–1.83)^a^	0.905
Dysphagia (absent)	1.79 (1,21–2.66)	0.004
Discrimination power of model: AUC (95 % CI): 0.713 (0.673–0.752)		
Calibration of model: Hosmer-Lemeshow test: *p* = 0.636		
Relative functional gain ≥35 %		
Diagnosis of respiratory disease (absent)	1.89 (1.32–2.71)	0.001
Functional capacity at admission		
Barthel index ≤10	ref.	
Barthel index 11–25	2.63 (1.67–4.15)	<0.001
Barthel index >25	5.36 (3.48–8.23)	<0.001
Discrimination power of model: AUC (95 % CI): 0.705 (0.659–0.750)		
Calibration of model: Hosmer-Lemeshow test: *p* = 0.978		
Rehabilitation Efficiency index ≥0.50		
Diagnosis of respiratory disease (absent)	1.72 (1.16–2.55)	0.007
Functional capacity at admission		
Barthel index ≤10	ref.	
Barthel index 11–25	1.79 (1.08–2.97)	0.025
Barthel index ≥26	2.60 (1.55–4.35)	<0.001
Dysphagia (absent)	1.92 (1.23–2.98)	0.004
Discrimination power of model: AUC (95 % CI): 0.689 (0.640–0.739)		
Calibration of model: Hosmer-Lemeshow test: *p* = 0.891		

OR (95 % CI): Odds Ratio, Confidence Interval 95 %

^a^Ceiling effect

## Discussion and conclusions

Although prognostic factors affecting rehabilitation outcomes and RII scores are known and have been studied by others, clear guidelines on the use and choice of the available RIIs are lacking. The novelty of the present study is its focus on the indications for using these indices in geriatric patients. We would highlight the different prognostic factors identified in the three different RIIs studied, and specifically, their advantages and limitations in elderly patients.

All three RIIs were useful to evaluate changes in functional capacity; however, each uses different formulas that can be affected by certain variables but not by others (Tables 3 and 4). The AFG was associated with a high number of variables in both the PHSV and AHSV groups; most of these associations have been reported by other authors [9, 24, 25]. In addition, functional capacity at admission was inversely associated with AFG (Table 3). In other words, less functional capacity was related to higher AFG, and *vice versa*. This must be taken into account when analysing rehabilitation outcomes in patients with slight or moderate disability because, in these cases, the AFG is limited by the maximum possible score on the scale that is used (100 points in the case of the BI). For example, the maximum AFG that a patient with a BI-adm value of 80 points can achieve is 20 (the ceiling effect of a 100-point scale). Therefore, the use of AFG as the only parameter of rehabilitation effectiveness in patients with light-moderate dependency could underestimate the results [9, 10, 27].

Given that the BI is not a continuous interval scale, AFG calculation using this index can be affected because a change in score does not have a consistent meaning in all patients. A 20-point AFG score is excellent for a patient who had lost 20 points prior to hospital admission; it represents 100 % recovery. However, it could be a disastrous outcome for a person who had lost 80 points from their BI-premorbid score. On the other hand, AFG only reflects an increase in “points on the scale” but does not provide any information about the maximum possible score on that scale; therefore, it is impossible to know whether the patient has achieved the maximum possible improvement. Therefore, some authors have suggested that AFG only indicates rehabilitation effectiveness in “reducing the level of dependency” [25].

Finally, it is difficult to establish the detected level of change in BI scores (AFG) that can be considered clinically relevant. Some authors have shown that a 20-point increase in the AFG has a favourable prognostic effect on long-term functional capacity and survival [12]; this limit has been used in other studies (including our own) as the threshold for clinically significant change [12, 23–25]. For the REI, according to other authors [17], ≥0.50 points can be considered clinically significant.

The RFG was significantly related with more factors from the AHSV group than from the PHSV, perhaps because the RFG minimizes the effect of prior conditions on rehabilitation outcome due to its reliance on previous functional capacity as the maximum potential to be reached. This reduces “the goal” to the level of the patient’s approximate situation before the hospital admission, independent of medical history, comorbidities or previous level of independence. At the same time, RFG shows the proportion of functional capacity that has been lost recently, due to the acute process that led to hospitalization, without taking into account whether the disability existed before hospital admission. In other words, it shows the portion of disability that is potentially reversible. On the other hand, the RFG has no “ceiling effect”; AFG values decrease as BI-adm increases, while RFG increases according to BI-adm (Table 3). Finally, patients in Group IV, who achieved complete recovery (RFG = 100 %), had the same mean AFG values as others (Group III) who did not reach the same level of recovery but did recover more than one third of their lost functional capacity (RFG = 35–99 %) (Table 2). This suggests a lack of information from the AFG about the extent to which the patient fully recovers premorbid functional status. Taking all of these considerations into account, we conclude that RFG contributes more valuable –and more qualitative– information to evaluating the rehabilitation process in elderly patients, who often present with chronic diseases and prior disability.

Our study has several limitations that should be taken into account. A RFG score ≥35 % has been described as a threshold of clinical rehabilitation effectiveness [10, 17, 23] and no higher distribution has been published to date. In our sample, however, most of the patients (72.9 %) achieved a higher RFG, and a substantial percentage (27.5 %) scored 100 %; therefore, the authors followed clinical criteria to establish the RFG score groups in the present sample. As this stratification had not been previously described and could lead to non-homogeneous groups, this could be considered a limitation of our study. In addition, RFG values can be difficult to compare between studies because they vary according to whether or not the values were adjusted for the BI-premorbid score using the theoretical maximum score (100 points) or the actual (individualized) BI-premorbid score. Obviously, this has the potential to generate confusion in attempts to generalize the use of this parameter and may be a confounding factor for comparative analysis.

Mathematically, it is debatable whether a variable (BI-adm) already included in the formula of the same outcome variable (RFG) can be considered as a predictor. Nonetheless, we must take into account the RFG’s inclusion of the BI-disch score in addition to BI-adm and BI-premorbid; inclusion of BI-adm as an independent variable was based on a reasonable suspicion (supported by the modelling) that a patient’s capacity for recovery could be influenced by the functional status at admission. It is logical to think that patients in a worse initial functional state would have less capacity for recovery. For this reason, we included the BI-adm in the model as an independent variable. A new assessment after functional capacity has improved could be of interest to test the power of these RII scores in comparison with other performance scales, such as Tinetti score, Short Physical Performance Battery or Gait Speed.

REI was related to nine variables, four from the PHSV group and five from the AHSV group. In the present study, mean REI was 2.0, which is higher than that reported in other similar settings [16, 17, 24, 28]. A possible explanation is the low prevalence of neurological diagnoses in our study population (Table 1). It has been demonstrated that patients with neurological disorders have lower REI values than patients with orthogeriatric or other diagnoses [9, 28]. Otherwise, REI is clearly influenced by length of stay, which was shorter in the present study than in others [24, 25]; this could be due to the inclusion criterion requiring that participants meet conditions amenable to discharge within two weeks; these individuals may have had better prognosis than other study populations. Another aspect that could have contributed to the high REI observed is that the functional status of patients admitted to an acute care unit may be underestimated because of barriers such as catheters, intravenous lines, and bed-rails that are prevalent in conventional hospitalization. In the first days after patients are transferred to a geriatric rehabilitation unit, rapid functional improvement tends to occur as hospital care can be combined with integrated care designed to promote autonomy and minimize dependency (common dining, social spaces, etc.). In the hospital setting, REI value is highly dependent on the length of the hospital stay. This must be taken into account because length of stay can be influenced by comorbid conditions that may interrupt rehabilitation [16, 29] and by individual variables (social, personal, or family issues) that are not directly related to rehabilitation therapy [25, 28]. To avoid this potentially confounding factor, some authors have suggested that REI should be calculated on the basis of the entire period during which the patient receives rehabilitation therapy. Using this approach, the denominator is the number of days of rehabilitation therapy, from start to finish, regardless of where it is provided [9]. In our sample, BI-adm had a mean value of 28 points, similar to that obtained by other authors in similar settings [24, 25, 29].

As shown in Table 5, PHSV (comorbidity and Lawton index) were only related with AFG and not with the rest of the RIIs. Similarly, two of the AHSV variables (absence of dysphagia and absence of respiratory diagnosis) were related to both AFG and REI in multivariate analysis. Evidence of the AFG ceiling effect was obtained in the BI-adm >50 group. Only two variables were predictors of functional recovery (RFG ≥35 %): functional capacity at admission and the absence of a diagnosis of respiratory disease (both from the AHSV group). Only BI-adm was related to all three RIIs. The BI cut-off values that had predictive value reflect severe dependency. This may be due to the study population, which was severely incapacitated upon admission but had a better prognosis than populations in other studies, which would reduce the cut-off values used to discriminate worse functional prognosis (BI ≤10). A large proportion of the patients came from an acute care geriatric unit, where respiratory diagnoses are very frequent; a poor functional prognosis for these patients has been described [30], and supports our observation of a better functional recovery in the absence of respiratory disease.

Finally, we would note that AFG was the parameter that was correlated with the greatest number of prognostic variables, but has the disadvantage of a ceiling effect and also does not provide evidence of the final functional outcome of rehabilitation therapy. REI is highly conditioned by the mean length of stay, which could be influenced by multiple factors that do not depend on the rehabilitation process. RFG seems to be more associated with variables that reflect health status at admission (severity of the recent acute process) and less affected by previous health status. Also, RFG provides more precise information about the degree to which a patient returns to his or her premorbid status. A future implication of the present study is that these considerations should be taken into account when selecting parameters to determine rehabilitation effectiveness in elderly patients.

## Additional file

Additional file 1:
**STROBE Statement—Checklist of items that should be included in reports of cohort studies.**

## Abbreviations

AFG: absolute functional gain
AHSV: admission health status variables
BI: Barthel index
BI-adm: Barthel index at admission
BI-disch: barthel index at discharge
BI-premorbid: barthel index premorbid
PHSV: premorbid health status variables
RFG: relative functional gain
REI: Rehabilitation efficiency index
RIIs: Rehabilitation Impact indices
RII: Rehabilitation Impact index
