# Supplementary material for: Three measures of physical rehabilitation effectiveness in elderly patients: a prospective, longitudinal, comparative analysis
Source: BMC Geriatr. 2015 Oct 29;15:142. doi: 10.1186/s12877-015-0138-5 (PMC4627405; doi:10.1186/s12877-015-0138-5)
Supplement: Additional file 1: — STROBE Statement—Checklist of items that should be included in reports of cohort studies. [file 12877_2015_138_MOESM1_ESM.doc]

STROBE Statement—Checklist of items that should be included in reports of ***cohort studies***

|  | Item No | Recommendation |
| --- | --- | --- |
| **Title and abstract** | 1 | (*a*) Indicate the study’s design with a commonly used term in the title or the abstract (Title page, Abstract)  Page 1: Title  Page? Abstract |
|  |  | (*b*) Provide in the abstract an informative and balanced summary of what was done and what was found  Page Abstract. |
| Introduction | | |
| Background/rationale | 2 | Explain the scientific background and rationale for the investigation being reported  Pages 3-4: |
| Objectives | 3 | State specific objectives, including any prespecified hypotheses    Page 3 (final paragraph) - top of Page 4: prespecified hypotheses.  Page 4 (end of continued paragraph): specific objectives. |
| Methods | | |
| Study design | 4 | Present key elements of study design early in the paper  Pages 4-5 |
| Setting | 5 | Describe the setting, locations, and relevant dates, including periods of recruitment, exposure, follow-up, and data collection  Pages 4-6 |
| Participants | 6 | (*a*) Give the eligibility criteria, and the sources and methods of selection of participants. Describe methods of follow-up  Pages 4-5 (Note: there was no follow up after discharge.) |
|  |  | (*b*) For matched studies, give matching criteria and number of exposed and unexposed  Not applicable: this was not a matched study |
| Variables | 7 | Clearly define all outcomes, exposures, predictors, potential confounders, and effect modifiers. Give diagnostic criteria, if applicable  Pages 5-6: data collection  Pages 6-7: outcomes  Exposures, predictors, potential confounders, effect modifiers and diagnostic criteria are not applicable to our study. |
| Data sources/ measurement | 8* | For each variable of interest, give sources of data and details of methods of assessment (measurement). Describe comparability of assessment methods if there is more than one group  Pages 6-7: calculation and cut-off points of outcomes measures; data source for all variables |
| Bias | 9 | Describe any efforts to address potential sources of bias  Pages 6 (second paragraph) - page 7, efforts to minimize errors and bias (also included in Table 2 legend, page 22) |
| Study size | 10 | Explain how the study size was arrived at  Pages 5 and 8 |
| Quantitative variables | 11 | Explain how quantitative variables were handled in the analyses. If applicable, describe which groupings were chosen and why  Page 5, 6, 7: variables, cut-off points and groups  Table 1, 2 and 3 (pages 20, 21 and 22): description of groupings |
| Statistical methods | 12 | (*a*) Describe all statistical methods, including those used to control for confounding  Page 7, Statistical Methods paragraph (also Table 2 legend, page 22) |
|  |  | (*b*) Describe any methods used to examine subgroups and interactions  Pages 6-7, 11-13; Table 2 and footnote, page 22 |
|  |  | (*c*) Explain how missing data were addressed  Pages 6 (undetermined values) and pages 21 and 24, Tables 1 and 3 (footnotes explain missing data) |
|  |  | (*d*) If applicable, explain how loss to follow-up was addressed  Not applicable |
|  |  | (*e*) Describe any sensitivity analyses  Not applicable |
| Results | | |
| Participants | 13* | (a) Report numbers of individuals at each stage of study—eg numbers potentially eligible, examined for eligibility, confirmed eligible, included in the study, completing follow-up, and analysed  Page 8, and footnotes to Table 1 and 3, pages 21 and 24. |
|  |  | (b) Give reasons for non-participation at each stage  Page 8 |
|  |  | (c) Consider use of a flow diagram  Not applicable |
| Descriptive data | 14* | (a) Give characteristics of study participants (eg demographic, clinical, social) and information on exposures and potential confounders  Page 8 and Tables 1 (page 20-21), 2 (page 22-23), 3 (page 24) and 4 (page 25) |
|  |  | (b) Indicate number of participants with missing data for each variable of interest  Page 8 and pages 21 and 24, footnotes to Tables 1 and 3 |
|  |  | (c) Summarise follow-up time (eg, average and total amount)  Not applicable, there was no specific follow up after discharge in our study. |
| Outcome data | 15* | Report numbers of outcome events or summary measures over time  Pages 8-9 and Tables 1, 2 and 3, page 21-25 |
| Main results | 16 | (*a*) Give unadjusted estimates and, if applicable, confounder-adjusted estimates and their precision (eg, 95% confidence interval). Make clear which confounders were adjusted for and why they were included    Page 7: Statistical section. and Page 26::Table 5 includes 95% confidence interval |
|  |  | (*b*) Report category boundaries when continuous variables were categorized  Pages 5-9 |
|  |  | (*c*) If relevant, consider translating estimates of relative risk into absolute risk for a meaningful time period  Not applicable |
| Other analyses | 17 | Report other analyses done—eg analyses of subgroups and interactions, and sensitivity analyses  Page 9: Statistical section.  Page 22: Table 2 |
| Discussion | | |
| Key results | 18 | Summarise key results with reference to study objectives  Pages 10 (discussion of results) and 14 (Conclusion) |
| Limitations | 19 | Discuss limitations of the study, taking into account sources of potential bias or imprecision. Discuss both direction and magnitude of any potential bias  Pages 11-12 |
| Interpretation | 20 | Give a cautious overall interpretation of results considering objectives, limitations, multiplicity of analyses, results from similar studies, and other relevant evidence  Pages 11-14. |
| Generalisability | 21 | Discuss the generalisability (external validity) of the study results  Pages 11-14 |
| Other information | | |
| Funding | 22 | Give the source of funding and the role of the funders for the present study and, if applicable, for the original study on which the present article is based  Page 15: No internal or external funding was received to support this research. |

*Give information separately for exposed and unexposed groups.

**Note:** An Explanation and Elaboration article discusses each checklist item and gives methodological background and published examples of transparent reporting. The STROBE checklist is best used in conjunction with this article (freely available on the Web sites of PLoS Medicine at http://www.plosmedicine.org/, Annals of Internal Medicine at http://www.annals.org/, and Epidemiology at http://www.epidem.com/). Information on the STROBE Initiative is available at http://www.strobe-statement.org.
